# Evaluation of l-arginine supplement on the growth rate, biofilm formation, and antibiotic susceptibility in *Streptococcus mutans*

**DOI:** 10.1186/s40001-022-00735-7

**Published:** 2022-07-02

**Authors:** Samaneh Vaziriamjad, Mobina Solgi, Farideh Kamarehei, Fatemeh Nouri, Mohammad Taheri

**Affiliations:** 1 Department of Oral Medicine, Dental School, Hamadan University of Medical Sciences, Hamadan, Iran; 2 Department of Medical Microbiology, Faculty of Medicine, Hamadan University of Medical Sciences, Hamadan, Iran; 3 Department of Pharmaceutical Biotechnology, School of Pharmacy, Hamadan University of Medical Sciences, Hamadan, Iran

**Keywords:** l-Arginine, *Streptococcus mutans*, Biofilm formation, Growth rate, Antimicrobial susceptibility

## Abstract

**Introduction:**

Bacteria associated with dental caries have a high ability to produce organic acids from dietary carbohydrates during growth and metabolism under acidic conditions. In contrast, many symbiotic bacteria produce ammonia through the arginine deiminase (ADS) system, which modulates the pH of the oral cavity. l-Arginine metabolism by ADS is a significant inhibitor in the progression of tooth decay. This study aimed to investigate the effect of l-arginine on growth, biofilm formation, and antibiotic susceptibility in *Streptococcus mutans*.

**Methods:**

In this study, the effect of l-arginine in different concentrations on the growth rate, antibiotic susceptibility, and inhibition of biofilm formation in *S. mutans* was investigated.

**Results:**

The bacterial exponential growth rate was enhanced by 100 μM l-arginine (*P* > 0.05). The growth inhibition zone diameter of CAZ, CTR, AMP, and AMC-Clav antibiotics was reduced after 24 h of exposure in the presence of various concentrations of l-arginine specifically at 100 μM. l-Arginine also enhanced biofilm development at 5 and 10 μM concentrations, but reduced it at 50 and 100 μM concentrations.

**Conclusion:**

According to the results of the present study, optimization of l-arginine concentration and its use as an adjunctive therapy or in combination with mouthwash or varnish is recommended to prevent oral caries.

## Introduction

Tooth decay is a complex disease that is influenced by a variety of factors. Acid is produced at the contact between the surface of the vulnerable tooth and dental plaque when sugar and carbohydrates are consumed in the diet. Gingival recession and root surface exposure function as supplemental or secondary components in the cariogenic process [1].

*Streptococcus mutans* (*S. mutans*) exist naturally in the human oral microbial flora and have receptors that improve adhesion to tooth surfaces. In adherent cells, the glucan sucrase enzyme in *S. mutans* is responsible for converting sucrose into dextran-based polysaccharides. As a result, causing sucrose to aggregate and form plaques. *S. mutans* use sucrose as a substrate to produce dextran via the enzyme dextransucrase (hexosyltransferase) [2, 3].

Biofilms are completely structured aggregates of physiologically and genetically diverse bacteria communities [4, 5] that use this feature to produce dental disease. The microbiota associated with dental caries and oral infections is acid-producing (acid-containing) flora linked with dental caries, such as *S. mutans* and specific Lactobacillus and Bifidobacterium, which increase the early probability of cavities [6, 7]. On the other hand, increasing the number of acid-tolerant bacteria (such as *Streptococcus gordonii*) can enhance tooth health [9]. Dental biofilm, in which bacteria colonize the surface of artificial tissues or implants, and are placed in the extracellular matrix of external polymers (polysaccharides and proteins) and their DNA [8, 9].

*S. mutans* constitutes 30% of plaque microflora and is a major cause of tooth decay in humans. This is related to the ability of bacteria to form biofilms, ferment multiple carbohydrates to organic acids, and acid growth and metabolism [10].

l-Arginine is a guanidine-containing essential amino acid and can promote protein solubility while also suppressing protein accumulation [11]. l-Arginine may influence cariogenic biofilms and it is mostly taken from the diet and host saliva is eaten by arginolytic bacteria (e.g., *S. gordonii*) in the oral cavity via the arginine deiminase (ADS) system, which produces ammonia as a byproduct. l-Arginine has the potential to improve oral health by increasing the pH of dental plaque, preventing biofilm formation, and disintegrating biofilms.

This study aimed to evaluate the effect of l-arginine on growth, biofilm formation, and antibiotic susceptibility in *S. mutans.*

## Material and methods

### Bacterial strains and culture conditions

*Streptococcus mutans* bacteria ATCC 35668 was obtained from the Pasteur Institute of Iran (Tehran, Iran). The l-arginine solution was prepared using a dissolution of l-arginine powder in water at 5, 10, 50, and 100 µM concentrations in a sterile condition. Culture media TSB was purchased from Merck company.

### Outgrowth curve

The effects of l-arginine treatment on the growth rates of bacteria were investigated. The standard concentration (0.5 McFarland) of bacterial suspension was inoculated in the TSB medium precisely and then divided into 2 sets as a control and the treated groups. For estimating the number of bacterial cells in a TSB medium, the turbidity of each group was measured using optical density (OD) in 625 nm absorption at different times exposures during 72 h using a UV–visible spectrophotometer (UNICO UV-2100).

### Biofilm production assay

Biofilm formation was evaluated by the microtiter plate method that was done triplicate. *S. mutans* (ATCC 35668) isolates were prepared and incubated at 37 °C overnight. A 0.5 McFarland standard microbial suspension was prepared; 200 µL of was added to each well and incubated for additional 16–18 h at 37 °C. Normal saline was used three times in the washing step, then to fix cells, 200 µL absolute ethanol (96%) was added to wells; the good contents were pulled after 15 min, and lets plate was dried at room temperature. The staining step was carried out by adding 200 µL of 2% crystal violet for 5 min. After the color was removed, 200 µL of 33 percent acetic acid was added to each well and incubated for 15 min at 37 °C; the optical density was then recorded using an ELISA plate reader at a wavelength of 595 nm. The following values were allocated for a definition of the biofilm formation: non-biofilm producer: OD ≤ 1, weak biofilm producer: 1 < OD ≤ 2, medium biofilm producer: 2 < OD ≤ 3, and strong biofilm producer: OD > 3 [5].

### Antimicrobial susceptibility testing

Antibiogram susceptibility testing was performed by disk diffusion method (Kirby–Bauer) on Müller–Hinton agar (Merck, Germany) based on the Clinical and Laboratory Standards Institute (CLSI 2020). Antibiotic disks used included ceftriaxone (CTR 30 µg), tetracyclines (TET 30 µg), amoxicillin + clavulanate (AMC + Clav 30 µg), ciprofloxacin (CIPR 5 µg), cefotaxime (CTX 30 µg), ampicillin (AMP 10 µg), vancomycin (VAN 30 µg), ceftazidime (CAZ 30 µg), amikacin (AMI 30 µg), doxycycline (DOX 30 µg) (ROSCO, Denmark).

### Statistical analysis

SPSS software (version 22) was used for statistical analysis. Chi-square analysis was used for comparisons between the capacity of biofilm production and antibiotic resistance.

## Results

### Outgrowth curve

**Fig. 1:**
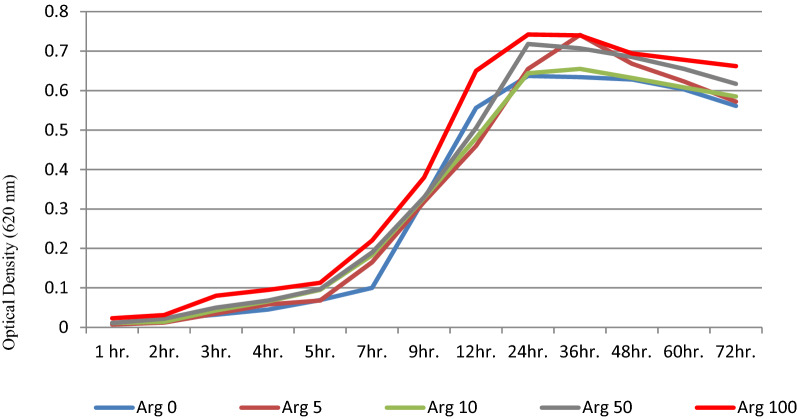
Growth curve diagrams of *S. mutans* after treatment with different l-arginine concentrations

The bacteria tend to proliferate faster after 2 h of exposure to 100 µM arginine, reaching the greatest level after 12 h, which is substantially different from the control group (*P* > 0.05). According to Fig. 1, it seems that the bacterial growth is dependent on the arginine concentration compared to the control group (Arginine 0) which the bacterial growth stages are at the lowest level.

Different from the control group (*P* > 0.05), according to Fig. 1, it seems that the bacterial growth is dependent on the arginine concentration compared to the control group (Arginine 0) in which the bacterial growth stages are at the lowest level.

### Biofilm production assay

**Fig. 2:**
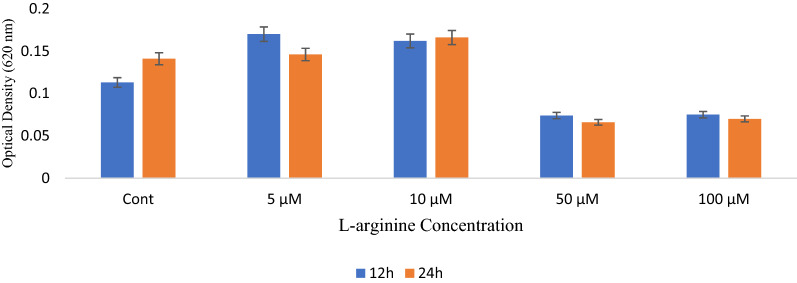
Biofilm formation ability of *S. mutans* after treatment with different l-arginine concentrations

According to the present study, bacterial exposure to 5 and 10 µM arginine compared to the control group at 12 and 24 h was associated with increased bacterial biofilm formation. However, bacterial biofilm formation at 50 and 100 µM concentrations was significantly reduced compared to the control group (*P* < 0.05), indicating the formation of arginine-dependent biofilm in bacteria. Bacterial biofilms were reduced when the arginine concentration was increased (Fig. 2).

### Antimicrobial susceptibility testing

**Fig. 3:**
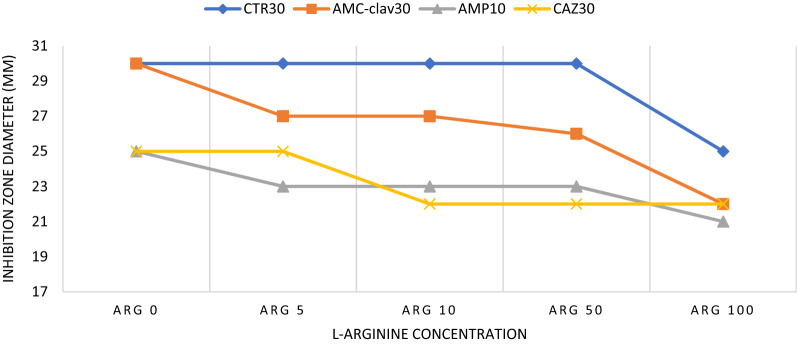
Antibiotic susceptibility of *S. mutans* after treatment with different l-arginine concentrations

The most antimicrobial susceptibility changes in CTR, AMC + Clav, AMP, and CAZ antibiotics were observed in different concentrations of arginine so that in CTR antibiotic, a decrease in the diameter of the growth inhibition zone was observed only in the concentration of 100 μM arginine after 24 h (Fig. 3). Although for the AMC + Clav antibiotic, the inhibition zone diameter was reduced in all concentrations of arginine, especially 100 μM. Changes in CAZ 30 antibiotic susceptibility were also seen after exposure to arginine concentrations of 10 μM and higher but were similar in AMP to AMC + Clav antibiotics. Although changes in the sensitivity of the mentioned antibiotics were observed in different concentrations of arginine, in general, a decrease in the inhibition zone diameter was observed, especially in the treatment with 100 μM arginine concentration in all of the antibiotics. On the other hand, in some antibiotics (TET, CIPR, CTX, VAN, AMI, and DOX), no remarkable changes in their antibiotic susceptibility were observed after 5 and 24 h of exposure to different concentrations of l-arginine (Table 1).

**Table 1:** Antibiotic susceptibility of *S. mutans* after treatment with different l-arginine concentrations (µM)

Exposure time	5 h					24 h				
Antibiotics	Arg 0 (Ctrl)	Arg 5	Arg 10	Arg 50	Arg 100	Arg 0 (Ctrl)	Arg 5	Arg 10	Arg 50	Arg 100
CTR	30	30	30	30	30	30	30	30	30	25
TET	30	30	30	30	29	30	30	30	29	27
AMC-Clav	30	30	30	30	30	30	27	27	27	22
CIPR	24	24	24	24	24	24	24	24	24	24
CTX	30	30	30	30	30	30	30	30	30	30
AMP	27	27	27	27	27	26	22	22	22	21
VAN	20	20	20	20	20	20	20	20	20	19
CAZ	25	25	25	25	25	25	25	22	22	22
AMI	13	13	13	13	13	13	13	13	13	13
DOX	30	30	30	30	30	30	30	30	30	30

## Discussion

*S. mutans* is the most common bacterium that forms biofilms on the tooth and is the leading cause of tooth decay. The mouth is the organism’s only known natural reservoir. *S. mutans* adheres to tooth surfaces through a variety of processes. Tooth decay is a microbial infectious disease of the tooth that results in the breakdown of the tooth's mineral components. Oral streptococci use the arginine deiminase system to metabolize arginine, which has several favorable benefits on oral health [12].

In a 2018 study, Nascimento and colleagues showed that arginine is converted to ammonia via the alkaline arginine deiminase (ADS) pathway, which counteracts the effects of the acid produced by bacterial glycolysis. Arginine deiminase in biofilm is also used to grow certain bacteria. Monitoring arginine metabolism through arginine deiminase can be an important criterion for assessing the risk of caries, and regular arginine inoculation into biofilms can be an effective treatment to prevent tooth decay. The results of this study were in line with our study so that the reduction of bacterial biofilm after 12 and 24 h at a concentration of 50 and 100 μM arginine was significantly determined and it will suggest that arginine can be used alone or in combination with other products to control caries [13]. Shivani Sharma and colleagues in a 2014 study showed that l-arginine can affect the adhesion of *S. mutans* biofilm in the oral cavity, it is the ability to adhere to the enamel surface [14]. The results of this study were controversial with our study so that it was found that the amount of biofilm formation is inversely related to l-arginine concentration and it seems that inhibition of biofilm formation occurs at concentrations above 50 μM. In 2016, Jinzhi He and colleagues showed that exposure to l-arginine may cause changes in the biofilm environment as bacteria grow under decay conditions. It appears that 1.5% l-arginine is clinically effective in modifying carcass biofilms through the production of alkalis by arginolytic bacteria. The data show new biological properties of l-arginine that affect biofilm matrix formation and microbial interactions associated with pathogenic biofilm proliferation [15].

Brinta Chakraborty et al., in a study that investigated the effects of arginine on the growth rate of *S. mutans*, stated that arginine negatively affects the ability of *S. mutans* to grow at 1.5% arginine supplementation. In contrast, our findings showed that at a concentration of 100 μM arginine (1.7%), the growth rate of bacteria was faster in the exponential phase, although no significant effects were observed [12].

Antibiotic overuse has been a major issue of drug resistance, resulting in a global worry about multi-antimicrobial resistant bacteria [16–18]. The oral cavity has recently been identified as a possible source of antibiotic resistance genes that can be passed down through oral biofilm bacteria via horizontal gene transfer [19]. In 2017, Xin Zheng et al. showed that the use of arginine can properly modulate the oral microbiota of people with caries. Concomitant use of arginine with fluoride can enrich the alkaline *Streptococcus sanguinis* and suppress *S. mutans*, and can therefore significantly delay the ability to form biofilms; Especially with arginine toothpaste, the mouth of people with active caries can be compared with ordinary people in terms of microbial structure, an abundance of common species, enzymatic activities of glycolysis and enzymes related to alkaline function. Contrary to our study, it reduced the growth of *S. mutans*, which can be caused by the simultaneous use of fluoride and l-arginine, but according to our study, it reduced the formation of biofilms [20].

A 2006 study by Borriello et al. showed that arginine can cause resistance to some antibiotics, which is consistent with our study [21]. In the present study, a significant reduction in antibiotic susceptibility was found only in the four antibiotics, which could be due to the following. As a result, it is critical to comprehend the chemical process behind this reaction. Different mechanisms characterized this phenomenon in earlier research on bacterial sensitivity:

(a) Antibiotic effectiveness can be attributed to interactions with water molecules in the aquatic environment [22]; (b) increasing the concentration of l-arginine alters the physicochemical characteristics and hydration ability of water molecules, as well as the solubility of antibiotics in the surrounding region [23]; (c) the type of peptidoglycan (PG) and the cell wall structure of bacteria are two factors that can influence antibacterial sensitivity. Gram-positive bacteria have thicker cell walls than Gram-negative bacteria [24]; (d) efflux pumps and ion channels located in the cell membrane play an important role in antibiotic uptake by the cell. l-Arginine concentrations may be capable of changing the channels and pumps and the duration of opening time will increase [25]; (e) the last factor that can influence the sensitivity of bacteria after exposure to substantial agents is the antibiotic structure. The charge, size, or hydrophilicity of the antibiotics can alter after being exposed to l-arginine concentration [26].

## Conclusion

According to the results of the present study, the effect of l-arginine in different concentrations on the growth of biofilm formation and antibiotic susceptibility of *S. mutans*, optimization of l-arginine concentration and its use as an adjunctive therapy or in combination with mouthwash or varnish is recommended to prevent oral caries.

## Data Availability

Not applicable.
